# A South American Prehistoric Mitogenome: Context, Continuity, and the Origin of Haplogroup C1d

**DOI:** 10.1371/journal.pone.0141808

**Published:** 2015-10-28

**Authors:** Mónica Sans, Gonzalo Figueiro, Cris E. Hughes, John Lindo, Pedro C. Hidalgo, Ripan S. Malhi

**Affiliations:** 1 Departamento de Antropología Biológica, Facultad de Humanidades y Ciencias de la Educación, Universidad de la República, Montevideo, Uruguay; 2 Department of Anthropology, University of Illinois, Urbana, Illinois, United States of America; 3 Carl R Woese Institute for Genomic Biology, University of Illinois, Urbana, Illinois, United States of America; University of Perugia, ITALY

## Abstract

Based on mitochondrial DNA (mtDNA), it has been estimated that at least 15 founder haplogroups peopled the Americas. Subhaplogroup C1d3 was defined based on the mitogenome of a living individual from Uruguay that carried a lineage previously identified in hypervariable region I sequences from ancient and modern Uruguayan individuals. When complete mitogenomes were studied, additional substitutions were found in the coding region of the mitochondrial genome. Using a complete ancient mitogenome and three modern mitogenomes, we aim to clarify the ancestral state of subhaplogroup C1d3 and to better understand the peopling of the region of the Río de la Plata basin, as well as of the builders of the mounds from which the ancient individuals were recovered. The ancient mitogenome, belonging to a female dated to 1,610±46 years before present, was identical to the mitogenome of one of the modern individuals. All individuals share the mutations defining subhaplogroup C1d3. We estimated an age of 8,974 (5,748–12,261) years for the most recent common ancestor of C1d3, in agreement with the initial peopling of the geographic region. No individuals belonging to the defined lineage were found outside of Uruguay, which raises questions regarding the mobility of the prehistoric inhabitants of the country. Moreover, the present study shows the continuity of Native lineages over at least 6,000 years.

## Introduction

The first study of ancient populations of Uruguay was based on a short segment of the hypervariable region I (HVRI) of the mitochondrial genome in five individuals [1]. Three individuals carried haplogroup C, one carried haplogroup B, and one could not be determined. Unexpectedly, two C1 individuals shared, besides the diagnostic mutations related to the haplogroup in the segment 16192–16355, a transition at nucleotide position (np) 16288C. This shared substitution was not given due attention until the analysis of the Charrúa Indian chief Vaimaca Perú (ca. 1780–1831), whose mtDNA showed the same mutation associated to haplogroup C1 [2]. According to historic sources on Uruguayan national identity, the Charrúa ethnic group was believed to be exterminated around the mid-19^th^ century [3]. However some years ago Acosta y Lara [4] identified several descendants of another Charrúa chief, named Sepé, demonstrating he had escaped the genocide. At present, the remains of Vaimaca Perú are the only recognized Charrúa Indian remains whose DNA have been analyzed. The shared 16288C mutation among the two ancient and one historic individuals suggested the possibility that living individuals may also share the mutation, and we set out to analyze HVRI sequences from contemporary Uruguayan individuals, with the aim to investigate the continuity of the prehistoric populations to the present, and particularly, to infer a possible relationship with Charrúa descendants [2].

Three out of 15 mtDNAs sequences of living Uruguayans carried diagnostic mutations of haplogroup C1d previously described in [5] had a mutation at np 16288C]. These three individuals lacked mutation at np 7697A, which initially defined haplogroup C1d [6,7], as well as Phylotree builds 1 to 6 (27 August 2008 to 28 September 2009) [8] but was later related to C1d1 [9,10], and Phylotree build 7 (10 November 2009) and following editions [8]. In a subsequent analysis, the complete mitogenome of one of those three Uruguayan individuals, as well as the whole HVRI of the two prehistoric individuals, was sequenced. The coding region mutations found in the first modern individual were also verified in the other two [2]. The study confirmed a lineage identified as belonging to haplogroup C1d (HVRI diagnostic pattern for this haplogroup: 16051G, 16223T, 16298C, 16325C, 16327T) characterized by 16288C as well as 194T, 16140C and 12378T, mutations recently used to define subhaplogroup C1d3 (Phylotree build 16, 19 February 2014 [8]).

However, some questions set out in the previous paper [2] are still unresolved, as how far back into ancient times we can trace each mutation found in the modern lineages defined as subhaplogroup C1d3, the chronological order of appearance of these mutations, the relationship of subhaplogroup C1d3 to other subhaplogroups C1d, and the present and ancestral geographical distribution of the subhaplogroup C1d3. In this article we continue studying this subhaplogroup deepen the analysis with the inclusion of a complete ancient mitogenome in an attempt to clarify the ancestral characteristics of subhaplogroup C1d3 and its phylogenetic relationships and completing the mitogenomes of four individuals partially studied in the paper published before [2]. Moreover, we used the distribution of C1d3 haplotypes to infer the peopling of the region of the Río de la Plata basin.

## Material and Methods

### The archaeological site

The mound CH2D01-A is the larger (1.4 meters high) of the two mounds that compose an archaeological site located in the department of Rocha, eastern Uruguay, near the Brazilian border. The archaeological site was excavated during 1986 and 1987, having the research permissions by the land owner and by the Comisión del Patrimonio Cultural de la Nación, Uruguay. During the mound excavation, primary and secondary burials of at least 21 Individuals were identified [11,12]. Its deepest archaeological level was dated to 2,090±90 YBP (KR139) and the most recent to 340±115 YBP (AC1199), while the oldest skeleton (named CH2D01-20) was dated to 1,610±46 YBP (AA 81800) and the most recent at 220±50 YBP (URU0014) [2,13]. In all cases, individuals buried are more recent than the archaeological level where the burial was recovered. The site corresponds to a complex, territorially circumscribed hunter-gatherer society [14], for which the practice of horticulture has been proposed based on the presence of domesticated crops in other archaeological site from the same region and similar chronology [15]. Despite the recent dates of the upper level, no evidence of Native-European contact was found. The ancient individual selected for mitogenome analysis, CH2D01-20, is a middle-aged adult female buried in the lowermost stratigraphic level of the mound.

### Modern individuals

Four complete mitogenomes from living individuals were analyzed. One of them, a Basque-descendant female from Trinidad in central Uruguay (B11), was published previously (GenBank accession number JQ701741 [2]). Two others had been partially published, as only small fragments of the mitogenome were assayed.

**Fig 1:**
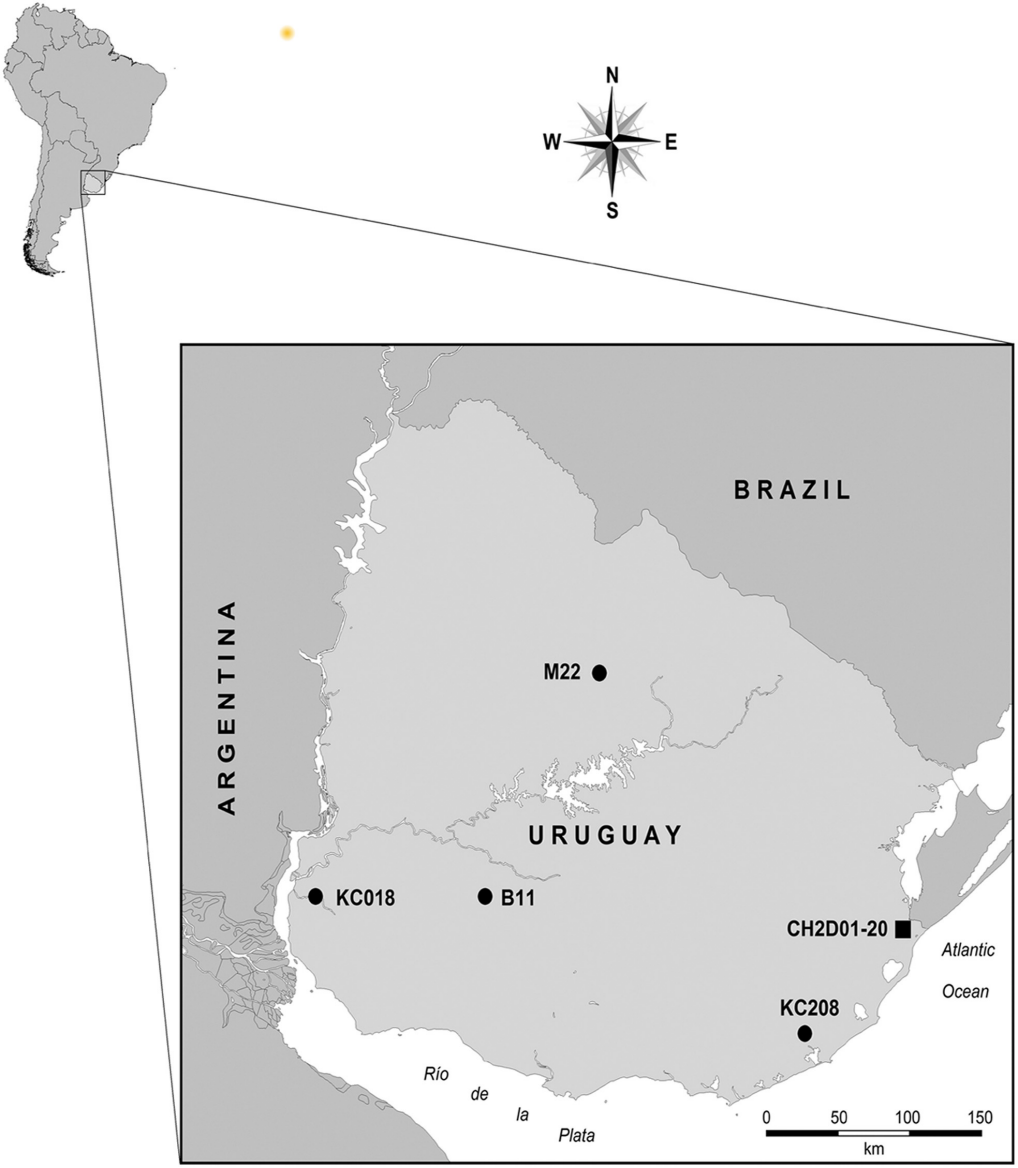
Map showing the location of the Uruguayan samples belonging to subhaplogroup C1d3 with the five complete mitogenomes used in this study. The locations of the living individuals (circles) correspond to their mothers’ birthplace. The archaeological site where the prehistoric sample was recovered is marked with a square.

These are two healthy females participating as controls in a project related with the study of breast cancer [16]: KC018, born in Montevideo but whose mother was born in Mercedes, western Uruguay, and KC208, from Rocha, in the east, approximately 100 km away from the CH2D01 site. We also investigated another individual, M22, a male whose HVRI had been analyzed years before [17]; he was born in Melo but his mother was from Tacuarembó, both cities located in the Northeast (Fig 1). Besides the birthplace of the mothers of the individuals, some information about maternal origins obtained from the interviews was proven to be likely erroneous: two grandmothers’ origins were indicated to be from Spain, which contrasts with the results obtained here.

### Ethical aspects

Uruguayan legislation does not require permission to study ancient remains; despite this, organizations of Charrúa descendants are informed about the studies. Modern individuals that participated in these studies, both related to ancestry analyses, provided written informed consent. The studies were approved by the Ethics Committee of the College of Humanities and Educational Sciences (University of the Republic, Uruguay) for individuals B11 and M22, and by the University of Chicago (USA) and the College of Medicine (University of the Republic, Uruguay) for KC018 and KC208.

### Ancient DNA extraction, preparation of genomic library, mtDNA enrichment, Illumina sequencing and bioinformatics analysis

The ancient DNA analysis reported here was performed to increase the resolution of the HVRI analysis carried out in previous work that defined the characteristics and minimum time span of haplogroup C1d3 [2]. A new extraction of DNA from CH2D01- 20 was performed at the Malhi Molecular Anthropology Laboratory University of Illinois at Urbana-Champaign (USA) using a mandibular second molar. The extraction and library build were conducted in the ancient DNA lab clean-room facility. Contamination controls were used with every DNA extraction and PCR setup in order to detect any contamination from reagents. A series of negative controls are routinely performed in the ancient DNA lab. The library was constructed using the New England Biolabs Ultra Kit for Illumina (E7370S, Ipswich, MA) following the manufacturer’s protocol with the following modifications. DNA fragmentation was not performed. DNA purifications were done using the MinElute Reaction Cleanup Kit (Qiagen, Valencia, CA). Library amplification was done in two steps. The first round of amplification utilized the kit’s reagents and protocol with 12 cycles of (10s at 98°C, 30s at 65°C and 30s at 72°C). For the second round, we achieved a sufficient DNA concentration for the mitochondria enrichment (~500ng), without excessive amplification, by creating 4 PCR reactions from the initial amplified product and then pooling them before using a Qiagen MinElute PCR Clean-up kit. For the second PCR, we created a 50 μl reaction, utilizing 0.2μM of primers P5 (5’- AATGATACGGCGACCACCGA-3’) and P7 (5’- CAAGCAGAAGACGGCATACGA-3’), 5μl from the initial PCR, 25μl of Phusion® High-Fidelity PCR Master Mix with HF Buffer (New England Biolabs, Ipswich, MA), 3% DMSO (New England Biolabs, Ipswich, MA), 0.2mg/ml BSA (New England Biolabs, Ipswich, MA). PCR conditions were as follows: 4min at 98°C, 10 cycles of (10s at 98°C, 30s at 62°C and 30s at 72°C), with a final extension at 72°C for 10min. Library fragment sizes were confirmed via a BioAnalyzer High Sensitivity assay to be above 130bp. A target enrichment of the mitochondrial genome was then performed on the amplified library using MYcroarray (Ann Arbor, MI) customized target enrichment kit following a target enrichment protocol modified for ancient DNA, as in [18]. A final post-enrichment amplification was performed for 15 cycles. The post-enrichment amplified product was then quantified using qPCR and submitted to High-Throughput Sequencing Division of the W.M. Keck Biotechnology Center at the University of Illinois Urbana-Champaign.

Raw data from the Illumina HiSeq 2000 platform was analyzed with CASAVA 1.8.2. In order to limit contamination that may have been introduced after the clean room library-building step, any reads that did not exhibit the exact index sequence were discarded. Adapter sequences were trimmed using AdapterRemoval [19] with a minimum length of 25 nps. Sequence reads were mapped to the human mitochondrial genome revised Cambridge reference sequence using Bowtie2 2.1.0 [20] with a local realignment option and a seed set to 1000. Duplicate reads were filtered based on mapping positions (-rmdup -s) using the SAMtools package 0.1.18 [21]. SNPs and INDELs were called using the SNVer package 0.4.1 [22]. SNP quality thresholds were set with a haploid model, a read depth of 20, base quality of 30, a nucleotide quality of 20, and alternate allele ratio of 0.9.

DNA damage (type I and type II) was assessed by comparing T–C/G–A and C–T/A–G transitions, respectively using MapDamage 2.0 [23]. A specific pattern of DNA damage has been identified in other ancient DNA studies [24–25]. These studies show a pattern of increased type II DNA damage at the beginning and end of degraded DNA fragments. An additional pattern can be inferred from an excess of purines at the genomic position before the sequencing start, which is indicative of strand fragmentation subsequent to post-mortem depurination [24]. We compared our results to other studies to assess if similar patterns of DNA damage were observed.

### Modern DNA extraction and sequencing

DNA extracts from individuals B11, KC018, and KC208 were available from previous studies [16,26]. A new DNA extraction from individual M22 was performed from hair at the Laboratory of Modern DNA of the Department of Biological Anthropology (University of the Republic, Uruguay) using the procedure described by Hidalgo et al. [27]. The mitogenomes of all four individuals were amplified by PCR using 27 overlapping fragments, as described previously [2]. (S1 Table). Fragments were sent for Sanger sequencing at the Molecular Biology Unit of the Institut Pasteur of Montevideo, using the same set of primers employed for amplification.

#### Sequence analyses

Sequences were aligned to the revised Cambridge Reference Sequence (rCRS; [28]) using Genedoc software version 2.7.000 [29]. Published and unpublished C1d complete mitogenome sequences from GenBank (S1 Text for details) were used for the construction of Median-Joining networks [30] using Network 4.6.1.2. The networks weres further processed using the maximum parsimony (MP) calculation [31] (see S1 Text).

#### Time to the most recent common ancestor (TMRCA) estimates

In order to explore the chronological relationship between subhaplogroup C1d3 and haplogroup C1d using mitogenome sequences, two estimates were computed for the TMRCA of each one. The first set of estimates was based on the ρ-statistic [32,33] between the sequences and the putative ancestral haplotype of each clade. The second set of estimates was carried out through coalescent Bayesian skyline plots (BSP [34]). The ages were computed using the mtDNA mutation rate calculated by Soares et al. [35] (S2 Text for details)

## Results

### Assessment of post-mortem DNA damage

Although several types of damage are found in ancient DNA [26], only two types of lesions are replicable–and thus amplifiable–by DNA polymerase in vitro. The first type, DNA fragmentation, is usually preceded by depurination [36], which has been found to leave a signature in the form of excess purines in the immediate vicinity of sequencing reads [24]. Miscoding lesions, in particular cytosine deamination, are seen as artifact C to T mutations, especially near the 5’ end of the read, and the corresponding G to A mutations near the 3’ end as the result of deamination in the complementary strand. All of these lesions can be seen in the output of the MapDamage script [23] used for the analysis (S1 Fig).

### Analysis of complete mitogenome sequences

**Fig 2:**
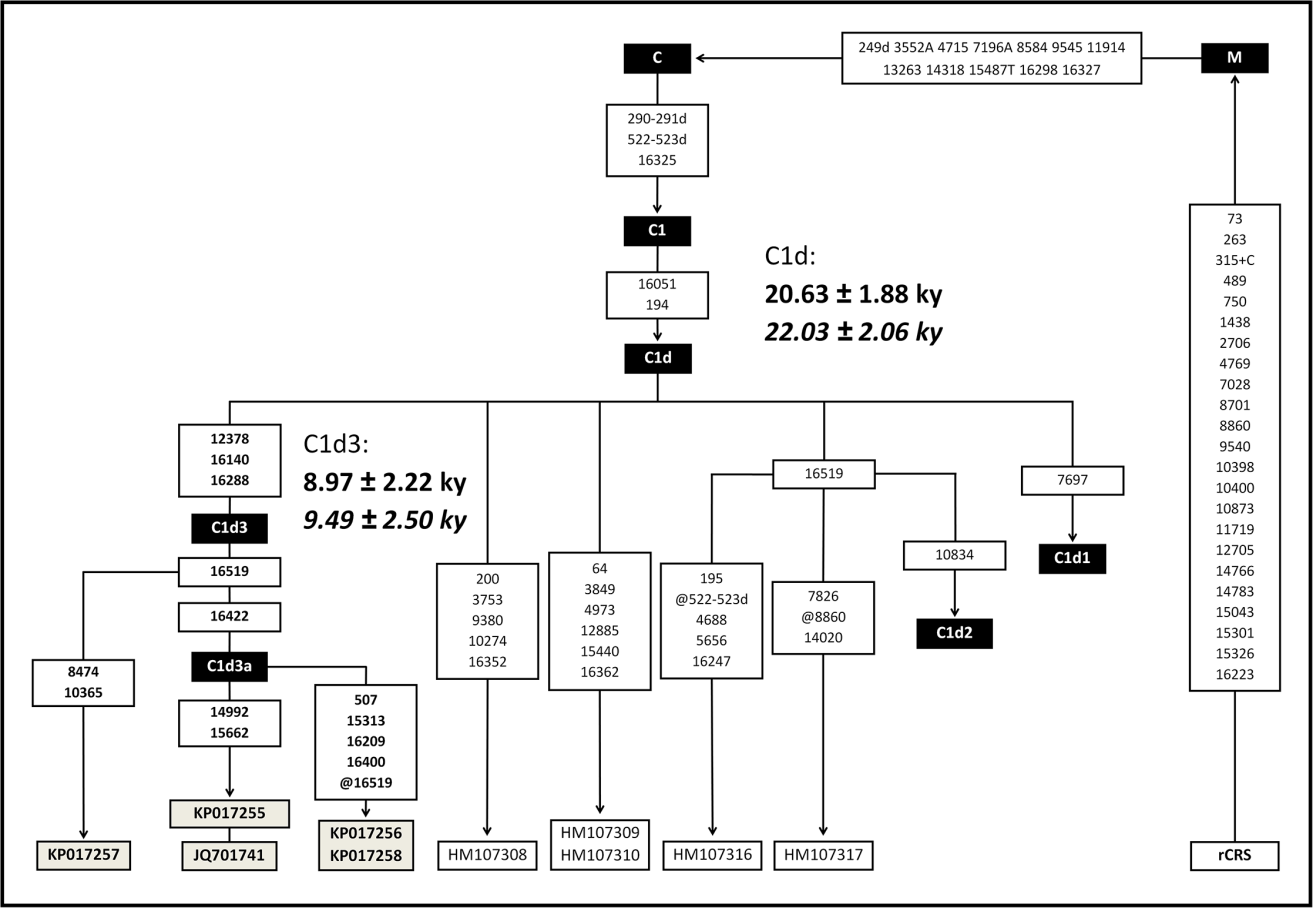
Evolutionary relationships between the C1d3 lineages, five other C1d sequences not belonging to subhaplogroupos C1d1 or C1d2, and position of the subhaplogroup within haplogroup C1d. Subhaplogroups are based on Phylotree build 16 [8]. GenBank accession codes of the sequences with the highest similarity to C1d3 (all previously published in [10]) are shown. Uruguayan sequences are inside grey boxes. Subhaplogroups C1d1 and C1d2 are collapsed for the sake of clarity. All mutations are included; they are transitions unless a base is explicitly indicated. The prefix @ designates reversions, while suffixes indicate transversions (to A, G, C, or T), indels (+, d). The age estimates (± 1SE) based on ρ are shown in bold and Bayesian estimates are in italic (see S2 Text for further details).

Complete mitogenome sequences corresponding to the ancient individual CH2D01-20 and living individuals M22, KC018 and KC208 were published in GenBank (accession numbers KP017255 (prehistoric), KP017258, KP017256, and KP017257 respectively), added to the previously published individual B11 (accession number JQ701741). All individuals share the diagnostic haplogroup C1 mutations as well as mutations 16051G and 194T which define haplogroup C1d. None of them have the mutation at np 7697 defining subhaplogroup C1d1 as well as the mutation at np 10834 defining subhaplogroup C1d2. Moreover, they all share mutations 12378T, 16140C, and 16288C. Mutation 16140C had not been found in the previous analysis of HVRI of CH2D01-20 [2], but was confirmed in the present study. Nine other mutations were also found in one or more individuals: 16422C (shared by CH2D01-20, B11, KC018 and M22), 14992C and 15662G (shared by CH2D01-20 and B11), 507C, 15313C, 16209C, 16400T (shared by KC018 and M22), and 8474A and 10365A (only found in KC208). Mutation at np16519 is shared by three individuals (KC208, CH2D01-20 and B11) and its absence in KC018 and M22 can be considered as a reversion in that line. The presence of 16422C shared by the four individuals mentioned above and belonging to two different lineages allows us to postulate a novel name, C1d3a, not mentioned in the last Phylotree build at the moment (build 16) [8]. The sequence of the prehistoric individual is identical to the modern individual B11, while sequences of individuals M22 and KC018 are identical to one another (Fig 2; for the complete list of mutations of each sample, see S3 Text). All mentioned positions are listed as differences to rCRS, while the presence of 16519T, coincidently with rCRS, is considered as a reversion.

Fig 2 also shows the evolutionary relationship between the Uruguayan and other complete mitogenomes belonging to paragroup C1d* (*sensu* [10]) which showed the greatest similarity with C1d3. These sequences are from Chihuahua, Mexico (sequence ID #4 in Perego et al. [10], GenBank ID HM107308), Kolla, Salta, Argentina (ID# 5a and 5b, HM107309 and HM107310 and Buenos Aires, Argentina (sequence ID #11, HM107316), and Boyaca, Colombia (sequence ID #12, HM107317), and were the unique C1d* not belonging to the previously described subhaplogroups C1d1 or C1d2 and having the mutation at np194 (np194C; see S1 Text). Sequences classified as C1d3 (all from Uruguay) are: KP017257 (KC208), KP017255 (CH2D01-20), JQ701741 (B11), KP017256 (KC018), and KP017258 (M22).Sequences classified as C1d1 (mutation at np 7697) and C1d2 (mutation at np 10834) were simplified and are showed as discontinued branches (Fig 2).

### TMRCA estimates

The means and 95% confidence intervals of the estimated time to the most recent common ancestors of subhaplogroup C1d3 and haplogroup C1d are detailed in Table 1. Each point estimate is located well outside each other’s confidence interval, thus reassuring the age estimates as representative of two distinct evolutionary events.

**Table 1:** TMRCA estimates for the putative common ancestors of haplogroup C1d and subhaplogroup C1d3.

Clade	Method	Age (years)	95% CI lower limit (years)	95% CI upper limit (years)
C1d	Rho	20,634	19,181	22,131
	BSP	22,033	18,347	26,335
C1d3	Rho	8,974	5,748	12,261
	BSP	9,487	4,837	14,304

BSP: Bayesian skyline plot. See Tables A and B in S2 Text for further details.

## Discussion

### On the characteristics, distribution and subhaplogroup assignment of C1d3 in mtDNA

None of the three mutations that characterize subhaplogroup C1d3 have been found in any other C1d control region sequence or mitogenome outside Uruguay. At present, one prehistoric and four living individuals, all from Uruguay, have been determined as belonging to subhaplogroup C1d3 [7], characterized by nps 12378T, 16140C and 16288C on a C1d background. It is also possible to add np 16519C in the subhaplogroup description, despite the fact that this position is considered a hotspot and the fastest in the mitochondrial genome [35,37,38]. This mutation is shared by three individuals (CH2D01-20, B11,KC018), while absent in the other two, likely due to a reversion. Other mutations are present only in some of the individuals: 16422C, defining a sublineage named C1d3a in this study is shared by individuals CH2D01-20, B11, KC018 and M22. A fifth individual (KC208) belongs to subhaplogroup C1d3 but not to C1d3a. The data presented here supports the existence of subhaplogroup C1d3 based on one mutation in the coding region and at least two in the HVR, while C1d is only characterized by HVR polymorphisms.

The subhaplogroup described for the five individuals considered in this study also seems to be shared by three other individuals whose mitogenomes could not be completely sequenced: a prehistoric individual also buried in CH2D01-A (clearly more recent than the prehistoric individual analyzed in this study), the historic (ca. 1780–1833) Charrúa Vaimaca Perú, and another living individual sampled in Montevideo [1,2,39]. The latter also carries the mutation at np 16209C (position 16400 was not sequenced). These three individuals all share the HVRI mutations that define subhaplogroupC1d3, and moreover, the living one probably belongs to the same haplotype within C1d3a as KC018 and M22. All this individuals have born in the Uruguayan territory and at the moment, no individuals out of Uruguay can be assigned to subhaplogroup C1d3.

When complete mitogenomes were considered, some sequences stand apart from Uruguayan subhaplogroup C1d3 (this study) as well as from subhaplogroups C1d1 and C1d2. These are one haplotype from Mexico, two haplotypes from Colombia and two from Argentina (one from Buenos Aires, its capital city and center of reception of migrants, and the other from the Kolla ethnic group in the northwest of the country) (Fig 2). The above mentioned haplotypes only share mutations at nps 16051 and 194, consistent with the current definition of haplogroup C1d [9], and consequently, a common origin can be traced back as far as the appearance of haplogroup C1d. No reversions to the ancestral state have been reported at np 16051 for any C1d sequences; on the other hand, reversions at np 194 seem to be frequent, but there are not observed in subhaplogroup C1d3.

### On the phylogeographic origin and age of C1d3 and its regional implications

As mentioned before, neither one nor two of the three diagnostic mutations that characterize subhaplogroup C1d3 in a C1d background have been found isolate. Consequently, at present it is not possible to establish the order of appearance of the mutations that define C1d3, even though the subhaplogroup has at least one sublineage. Moreover, and surprisingly, the entire evolution of this lineage might have occurred in Uruguayan territory, as it seems to be restricted to this country. The TMRCA of founding haplogroup C1d has been estimated at 15,200 ± 4,800 [9], 15,500 ± 5,232 [40] and 18,800 ± 2,800 YBP [10]. These estimates are significantly younger than our time estimates, which fall in the 18,300–26,300 year range. All estimates do however extend far beyond the first estimate of 9,200–9,500 YBP [7,41].

The C1d ages are also partially consistent with the published ages for haplogroup C1, although they can be deemed as roughly contemporaneous, with point estimates varying from 18,252 to 23,800 YBP [6,40,42] Another estimation [43] generated younger ages of 13,260 and 15,600 YBP, which seem too recent in view of C1d estimations.

The present date estimated for subhaplogroup C1d3 of 8,974 YBP, in the range of 4,387–14,304 YBP, overlaps the range estimated of 5,748–12,261 YBP for the TMRCA of South American C1d lineages [5]. The estimated date of C1d3 is in the range of those of C1d1 (15,500 ± 5,232 and 17,000 ± 1,700 YBP) and C1d2 (10,755 ± 4,484 and 11,700 ± 3,700 YBP) [9,40], respectively, despite its point estimate being younger. It has been noted that rho is subject to substantial systematic error [44], with a tendency to either under- or overestimate coalescence times depending on different demographic scenarios, but the concerns have been shown to be non relevant under specific circumpsances described in recent publications e.g. [45]. The underestimation of the age of subhaplogroup C1d3 seems to be minimal, in view of the small difference between the rho and the BSP estimates. In relative terms, estimates set the TMRCA of C1d3 at roughly half the TMRCA of C1d.

The chronology of the initial Uruguayan prehistoric peopling can contribute to the comprehension of the antiquity of subhaplogroup C1d3. Although a recent controversial study provides dates for human presence in the area between 27,000 ± 450 and 30,100 ± 600 ^14^C YBP (32,298–31,219 cal) [46], the bulk of the radiocarbon dates reveal an initial occupation of what is now Uruguay at around 10,400 to 12,600 YPB [47–52]. In view of this, the antiquity of C1d3 indicates that it could have originated in Uruguayan territory. It is believed that at least until 7,000 YBP inhabitants were highly mobile [53]. The apparent restriction of subhaplogroup C1d3 to the Uruguayan territory seems to contradict that mobility, especially considering that the CH2D01 site is close to the Brazilian border. Then, one possibility is that subhaplogroup C1d3 is not restricted to Uruguayan territory and the apparent lack of C1d3 in neighboring countries is due to the effect of sampling. If that were the case, then other individuals could be found in the future in areas adjacent to Uruguay. Alternatively, either the lineage is younger than proposed, or the mobility of early peoples was indeed not as high as supposed. As for the first possibility, the frequency of subhaplogroup C1d3 in the Uruguayan population is 0.7% [2], with a 95% confidence interval (using a normal approximation) of 0.094%-1.306%. Using this confidence interval for estimating required sample size, the mitochondrial genome of 1,060 individuals should be sampled in neighboring areas to find at least one subject carrying C1d3. In Argentina, at least 1128 individuals had been sampled as of 2011 [54]; in southern and southeastern Brazil, at least 1558 individuals have been studied as of 2014 [55–61]. Therefore, we find it unlikely that the absence of C1d3 in regions adjacent to Uruguay could be explained only by sampling error.

As for the second possibility, although there is evidence of long distance interaction networks linking Uruguay and the Argentinean Pampas during the Pleistocene/Holocene transition [62], several lines of evidence from the archaeological context of eastern Uruguay suggest that there was indeed a reduction in mobility in that particular area throughout the Holocene (e.g. [63]). Furthermore, the mound structures themselves have frequently been interpreted as territorial markers [64–66] that suggest a growing territorial circumscription, especially during the late Holocene. Although the archaeological phenomenon of the mounds in eastern Uruguay has a maximum antiquity of 5,400 years [13], the burials in these mounds comprise only the last 1,400 years of their time span [67], which leaves open the possibility of the mounds involving several populations during their existence. This would not necessarily result in total genetic isolation, but it would generate a political system based on kinship (as the one found, for example, in the Mapuche of Chile [68]) with a restricted flow of matrilineages. The fact that two individuals buried in the same mound belong to the same C1d3 lineage [2] is further–albeit circumstantial–evidence in favor of this possibility. Further studies are necessary to shed light on these aspects.

Recently, several haplogroups or subhaplogroups restricted to particular regions in South America have been defined. This is the case of B2j and B2k, identified in Venezuela [69], and D1j, D1g, B2i2 and C1b3, in the Southern Cone of South America [45,70–71]. Some of these are likely to be part of the first arrival in the region, about 15,000 YPB, while others (B2i2, C1b3) are more recent and related to local processes. According to de Saint Pierre et al. [70], these subhaplogroups might have evolved more recently in specific populations of the Southern Cone, a situation that can probably apply to C1d3.

Finally, we would like to emphasize that this study examines one of the first complete mitogenomes from an ancient individual from South America. This mitogenome provides information about the continuity through time in an enclosed region in Southeast South America. The information significantly increases the data about haplogroup C1d and shows the utility of using mitogenomes to understand evolutionary processes and population history of Native Americans at the regional level.

## Supporting Information

S1 Fig Output of the post-mortem damage assessment. a: relative frequencies of the four bases near (outside the grey frames) and at the 5’ and 3’ ends of the reads (grey frames). b: relative frequencies of T (in red) and A (in blue) near 5’ (positive) and 3’ (negative) positions of the reads.
(TIF)

S1 Table Primers used for the amplification and Sanger sequencing of the modern mitogenomes. (DOC)

S1 Text Published and unpublished sequences analyzed. (DOC)

S2 Text Time estimates of C1d and C1d3 carried out in this work. (DOC)

S3 Text Complete list of mutations found in the Uruguayan samples, respective to the rCRS (Andrews et al. 1999). (DOC)
