# Supplementary figures and images for: A South American Prehistoric Mitogenome: Context, Continuity, and the Origin of Haplogroup C1d
Source: PLoS One. 2015 Oct 28;10(10):e0141808. doi: 10.1371/journal.pone.0141808 (PMC4625051; doi:10.1371/journal.pone.0141808)

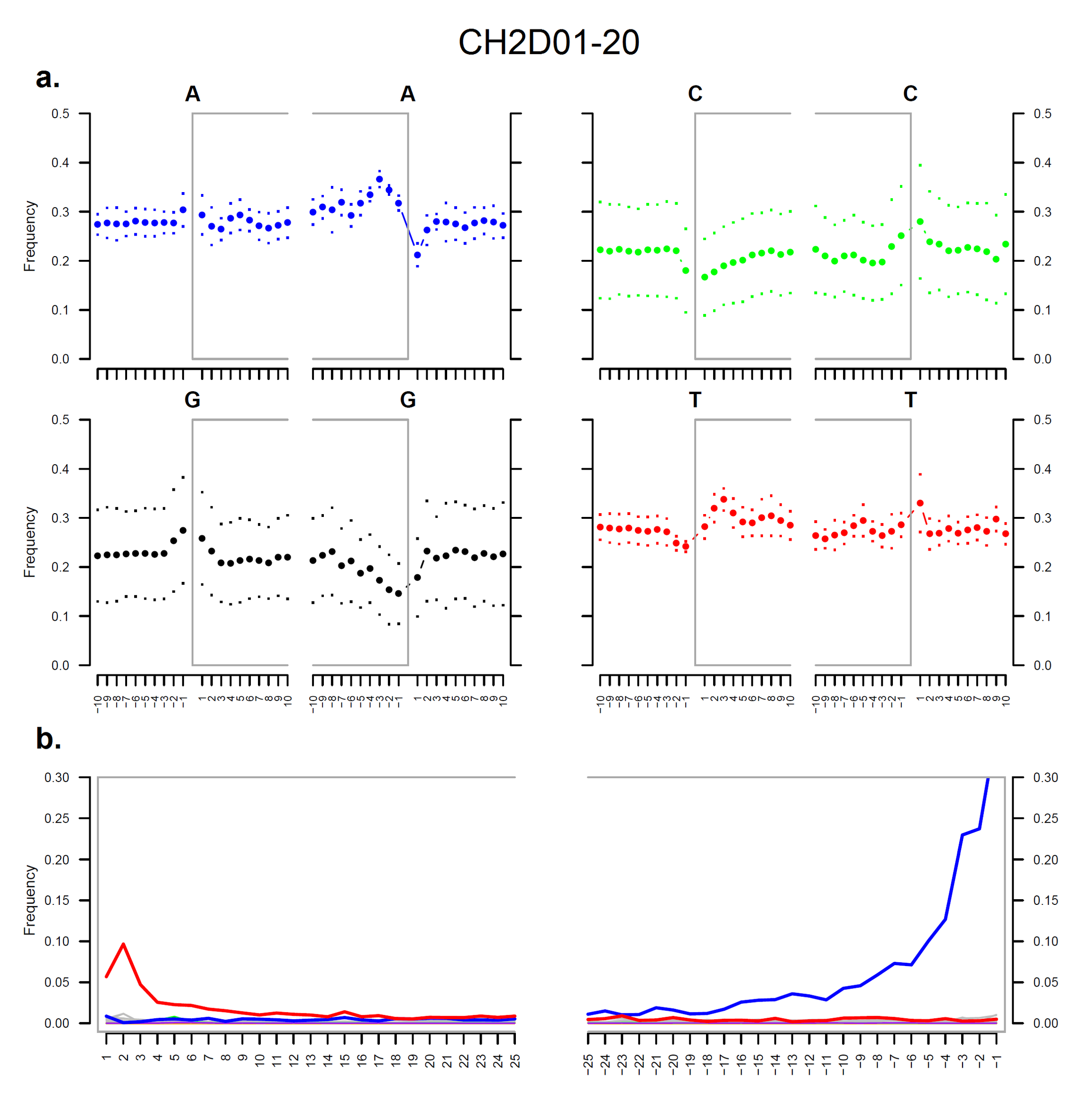

Supplement: S1 Fig — a: relative frequencies of the four bases near (outside the grey frames) and at the 5’ and 3’ ends of the reads (grey frames). b: relative frequencies of T (in red) and A (in blue) near 5’ (positive) and 3’ (negative) positions of the reads. (TIF) [file pone.0141808.s001.tif]
