# Supplementary material for: A South American Prehistoric Mitogenome: Context, Continuity, and the Origin of Haplogroup C1d
Source: PLoS One. 2015 Oct 28;10(10):e0141808. doi: 10.1371/journal.pone.0141808 (PMC4625051; doi:10.1371/journal.pone.0141808)
Supplement: S1 Table — (DOC) [file pone.0141808.s002.doc]

**S1 Table. Primers used for the amplification and Sanger sequencing of the modern mitogenomes.**

| **Fragment name** | **Forward Primer** | **Reverse Primer** | **Fragment Length (bp)** |
| --- | --- | --- | --- |
| MT1 | 29 [1] | 742 [2] | 758 |
| MT2 | 611 [3] | 1411 [3] | 841 |
| MT3 | 1245 [3] | 2007 [3] | 803 |
| MT4 | 1854 [3] | 2669 [3] | 856 |
| MT5 | 2499 [3] | 3346 [3] | 888 |
| MT6 | 3169 [3] | 3961 [3] | 833 |
| MT7 | 3592 [2] | 4489 [4] | 937 |
| MT8 | 4217 [2] | 4919 [2] | 742 |
| MT9 | 4462 [2] | 5229 [2] | 807 |
| MT10 | 5121 [2] | 6031 [3] | 951 |
| MT11 | 5855 [3] | 6642 [3] | 828 |
| MT12 | 6469 [3] | 7261 [4] | 833 |
| MT13 | 7148 [3] | 8095 [3] | 988 |
| MT14 | 7937 [3] | 8797 [3] | 901 |
| MT15 | 8216 [2] | 9086 [2] | 910 |
| MT16 | 8921 [2] | 9397 [3] | 517 |
| MT17 | 9230 [3] | 10127 [2] | 938 |
| MT18 | 9963 [2] | 10912 [5] | 977 |
| MT19 | 10814 [5] | 11472 [3] | 699 |
| MT20 | 11314 [3] | 12076 [3] | 803 |
| MT21 | 11948 [3] | 12772 [3] | 865 |
| MT22 | 12571 [3] | 13507 [3] | 977 |
| MT23 | 13233 [2] | 14268 [3] | 1076 |
| MT24 | 14000 [3] | 14998 [3] | 1039 |
| MT25 | 14856 [3] | 15978 [3] | 1163 |
| MT26 | 15811 [3] | 11 [4] | 810 |
| MT27 | 16210 [6] | 397 [5] | 794 |

Primer coordinates mark the nucleotide position of the 3’ end of the primer following the CRS [7]. Numbers between brackets indicate the reference of the primer sequence.

**References**

1. Vigilant L, Pennington R, Harpending H, Kocher TD, Wilson AC. Mitochondrial DNA sequences in single hairs from a southern African population. Proc Natl Acad Sci USA. 1989; 86: 9350-9354.

2. Martínez-Cruzado JC, Toro-Labrador G, Viera-Vera J, Rivera-Vega MY, Startek J, Latorre-Esteves M, et al. Reconstructing the population history of Puerto Rico by means of mtDNA phylogeographic analysis. Amer J Phys Anthrop. 2005; 128: 131-155.

3. Rieder MJ, Taylor SL, Tobe VO, Nickerson DA. Automating the identification of DNA variations using quality-based fluorescence re-sequencing: Analysis of the human mitochondrial genome. Nucleic Acids Res. 1998; 26: 967-973.

4. Sans M, Figueiro G, Hidalgo PC. A New Mitochondrial C1 Lineage from the Prehistory of Uruguay: Population Genocide, Ethnocide and Continuity. Hum Biol. 2012; 84: 287-305.

5. Sans M, Figueiro G, Ackermann E, Barreto I, Egaña A, Bertoni B, et al. Mitochondrial DNA in Basque Descendants from the City of Trinidad, Uruguay: Uruguayan- or Basque-like Population? Hum Biol. 2011; 83: 55-70.

6. Handt O, Krings M, Ward RH, Pääbo S. The Retrieval of Ancient Human DNA Sequences. Amer J Hum Genet. 1996; 59: 368-376.

7. Anderson S, Bankier AT, Barrell BG, de Bruijin MHL, Coulson AR, Droulin J, et al. Sequence and organization of the human mitochondrial genome. Nature. 1981; 290: 457-465.
