# Supplementary material for: A South American Prehistoric Mitogenome: Context, Continuity, and the Origin of Haplogroup C1d
Source: PLoS One. 2015 Oct 28;10(10):e0141808. doi: 10.1371/journal.pone.0141808 (PMC4625051; doi:10.1371/journal.pone.0141808)
Supplement: S1 Text — (DOC) [file pone.0141808.s003.doc]

**S1 Text: Published and unpublished sequences analyzed**

1. **Articles and databases used to analyze entire mitogenomes (in particular, search of haplogroup C1d diagnostic mutations plus mutations at nps 507, 8474, 10365, 12378, 14922, 15313, 15662, 16288, 16140, or 16422:** no C1 sequence with such mutations was found.

Genbank, http://blast.ncbi.nlm.nih.gov/. Accessed February 14th, 2015

Phylotree, mtDNA tree Build 16 (19 Feb 2014), http://www.phylotree.org/

Bodner et al. (2012) Genome Res 22:811-20

Cui et al. (2013). PLoS ONE 8:e66948.

De Saint Pierre et al. (2012a) PLoS ONE 7:e43486

de Saint Pierre et al (2012b) PLoS ONE 7:e51311

Dryomov et al (2015) Eur J Hum Genet Jan 7.

Fagundes et al. (2008) Am J Hum Genet 82:583–92

Garcia et al 2012 Am J Phys Anthropol 149:583-90

Ramallo et al. (2013) Am J Phys Anthropol 150:453-63

Rasmussen et al. (2014) Nature 506: 225-229.

Sans et al. (2012) Hum Biol 84:287-305

Taboada-Echalar et al. (2013) PLoS ONE 8: e58980

Tamm et al. (2007) PLoS ONE 2:e829.

1. **Other articles and databases used to analyze HVRI sequences (minimum region included np16051 to np 16380)** (search of diagnostic mutations of haplogroup C1d plus mutations at nps 16288 or 16140: no sequence was found).

EMPOP v3/R11, http://empop.org/modules/haplotype/ Accessed June 18th, 2015

Alves-Silva et al. (2000) Am J Hum Genet 67:444-61

Bobillo et al. (2010) Int J Legal Med 124:263–268

Bonilla et al. (2004) Am J Hum Biol. 2004 May-Jun;16(3):289-97.

Cabana et al. (2006) Am J Phys Anthropol 131:108-19

Cardoso et al. (2013) Forensic Sci Int Genet 7:e52-5

Catelli et al. (2011) BMC Genet 12:77

Corach et al. (2010) Ann Hum Genet 74:65-76.

Dornelles et al. (2004) Am J Hum Biol 16:479–88

Figueiro et al. (2011) PLoS One 6:e20978.

Gayà-Vidal et al. (2011) Am J Phys Anthropol 145:215-30

Ginther et al. (1993) EXS 67:211-9.

Kumar et al. (2011). BMC Evol Biol 11:293.

Lalueza et al. (1997) Hum Mol Genet 6:41-6.

Marrero et al. (2005) Am J Hum Biol 17:496–506.

Marrero et al. (2006) Am J Phys Anthropol 132:301-10

Marrero et al. (2007) Hum Hered. 64:160-71.

Martínez-Cortés et al. (2013) Am J Phys Anthropol. 151:526-37.

Mendizabal et al. (2008) BMC Evol Biol 21;8:213.

Merriwether et al. (1995) Am J Hum Genet 56:812-3.

Moraga et al. (2000) Am J Phys Anthropol 113:19-29.

Pagano et al. (2005) J Forensic Sci 50:1239-42

Prieto et al. (2011) Forensic Sci Int Genet 5:146–51

Ramallo et al. (2013) Am J Phys Anthropol 150:453-63

Sala et al. (2010) Hum Biol 82:433-56.

Salas et al. (2008a) Am J Hum Biol 20:584-91

Salas et al. (2008b) J Hum Genet 53:662-74

Sandoval et al. (2008) Hum Genet 126:521–31

Sans et al. (2002) Am J Phys Anthropol 118:33-44.

Sans et al. (2006) Am J Hum Biol 18:513-24.

Sans et al. (2011) Hum Biol 83:55-7.

Sans et al. (2012) Hum Biol 84:287-305

Sans et al. (2015) Am J Hum Biol 27:407-16.

Schmitt et al. (2004) Ann Hum Biol 31:87-94.

Torroni et al. (1993) Am J Hum Genet 53:563-90

Yang et al. (2010) Ann Hum Genet 74:525-38.

1. **C1d sequences used to build Network 1 (showed below) based on entire mitogenomes (N=62 incluiding rCRS). Mutations at np16519 were excluded, mutation path from rCRS is not shown.**

DQ282472 (C1-3-01)

DQ282473 (C1-3-02)

DQ282474 (C1-3-03)

EU095222 (WAIWAI16)

HM107306 (S-987389)

HM107308 (S-683498)

HM107309 (SA19)

HM107311 (Mst42)

HM107312 (Mst68)

HM107313 (Mst61)

HM107314 (Mst50)

HM107315 (Mst64)

HM107316 (ABS174)

HM107317 (S-635878)

HM107318 (S-632547)

HM107319 (S-914766)

HM107320 (S-677163)

HM107323 (SA27)

HM107324 (Di23)

HM107326 (Di22)

HM107327 (ABS228)

HM107328 (ARN116)

HM107329 (ABS299)

HM107330 (ACO388)

HM107331 (S-629812)

HM107332 (ABS284)

HM107334 (S-934519)

HM107335 (S-658745)

HM107336 (S-635687)

HM107337 (S-643125)

HM107338 (S-689881)

HM107341 (S-987167)

HM107349 (S-649798)

HM107352 (S-919735)

HM107354 (S-657487)

HM107355 (ABS155)

HM107356 (S-681199)

HM107363 (S-686788)

HM107364 (S-635448)

HM107365 (S-996796)

HM107366 (S-915151)

HM107367 (S-915473)

HQ012234 (MA007)

HQ012235 (MA053)

HQ012237 (MA065)

HQ012238 (MA072)

HQ012239 (MA079)

HQ012240 (MA096)

HQ012241 (MA147)

HQ012243 (MA180)

HQ012244 (MA182)

JQ701741 (B11)

JX413049 (XL003)

JX413050 (686285)

JX669313 (PE323)

JX669333 (PE807C)

KJ446441 (HGDP00706)

KJ923814 (Cree21)

KP017255 (CH2D01_20)

KP017256 (KC018)

KP017257 (KC208)

KP017258 (M22)

NC_012920 (rCRS)

Mutations at nps 194, 7697, 16311 have generated squares. Sequences in the upper part (over the black line) belong to subhaplogroup C1d1; sequences below the black line belong to all other subhaplogroups or are not classified. Names identifying the samples were taken from the corresponding GenBank record (see above).

Node colors: Green: Uruguayan sequences from living individuals. Blue: Uruguayan ancient individual (green + blue: C1d3). White: rCRS (not a scale regarding mutations). Yellow: all the others. Red: median vectors.

Network 1

1. **Network 2: C1d sequences excluding C1d1 sequences (N=21, plus rCRS).**

Mutations at np 16519 were excluded. Sample names refer to the names given in the Genbank records, as in Network 1.

Node colors: Green: Uruguayan sequences from living individuals. Blue: Uruguayan ancient individual (green + blue: C1d3). Purple: C1d2. Yellow: unclassified C1d (neither C1d1 nor C1d2) used in Figure 2. Black: unclassified C1d without 194. White: rCRS (mutations leading to rCRS not shown).

Codes: Mst: Colombian Mestizos; MA: Mexican Americans; ABS: Buenos Aires, Argentina; SA: Salta, Argentina; S-63587/8: Boyacá, Colombia; S-68349/8: Chihuahua, Mexico (see GenBank for more details), KC: Uruguayans; CH2D01: ancient individual from Uruguay (CH2D01-20).

Network 2

1. **Sequences used for Figure 2** (N=10 plus rCRS): are the same used in Network 2 without the ones without the mutations at np 194 (all Mexican Americans from Kumar et al. 2011 -) .

The remaining sequences (without mention the Uruguayan ones) were all published by Perego et al. (2010):

HM107308 (S-683498) – Chihuahua, Mexico (mixed population);

HM107309 and HM107310 (SA19 and SA51), Salta (Argentina), Kolla Native ethnic group;

HM107316 (ABS174) – Buenos Aires, Argentina (mixed population);

HM107317 (S-635878) – Boyacá, Colombia (mixed population);

KP017257 (KC208) – Uruguay;

KP017255 (CH2D01-20), Uruguayan, ancient, together with JQ701741 ( B11), Uruguayan;

KP017256 (KC018) together with KP017258 (M22), Uruguayans.
