# Supplementary material for: A South American Prehistoric Mitogenome: Context, Continuity, and the Origin of Haplogroup C1d
Source: PLoS One. 2015 Oct 28;10(10):e0141808. doi: 10.1371/journal.pone.0141808 (PMC4625051; doi:10.1371/journal.pone.0141808)
Supplement: S2 Text — (DOC) [file pone.0141808.s004.doc]

**S2 Text. TMRCA estimates for C1d and C1d3.**

1. **84 mitochondrial genome sequences used in the analysis**

| **Accession** | **Reference** | **Subhaplogroup** |
| --- | --- | --- |
| DQ282472 | [1] | C1d1 |
| DQ282473 | [1] | C1d1 |
| DQ282474 | [1] | C1d1 |
| EU095222 | [2] | C1d1 |
| HM107318 | [3] | C1d1 |
| HM107319 | [3] | C1d1 |
| HM107320 | [3] | C1d1 |
| HM107321 | [3] | C1d1 |
| HM107322 | [3] | C1d1 |
| HM107323 | [3] | C1d1 |
| HM107324 | [3] | C1d1 |
| HM107325 | [3] | C1d1 |
| HM107326 | [3] | C1d1 |
| HM107327 | [3] | C1d1 |
| HM107328 | [3] | C1d1 |
| HM107329 | [3] | C1d1 |
| HM107330 | [3] | C1d1 |
| HM107331 | [3] | C1d1 |
| HM107332 | [3] | C1d1 |
| HM107333 | [3] | C1d1 |
| HM107334 | [3] | C1d1 |
| HM107335 | [3] | C1d1 |
| HM107336 | [3] | C1d1 |
| HM107337 | [3] | C1d1 |
| HM107338 | [3] | C1d1 |
| HM107339 | [3] | C1d1 |
| HM107340 | [3] | C1d1 |
| HM107341 | [3] | C1d1 |
| HM107342 | [3] | C1d1 |
| HM107343 | [3] | C1d1 |
| HM107344 | [3] | C1d1 |
| HM107345 | [3] | C1d1 |
| HM107346 | [3] | C1d1 |
| HM107347 | [3] | C1d1 |
| HM107348 | [3] | C1d1 |
| HM107349 | [3] | C1d1 |
| HM107350 | [3] | C1d1 |
| HM107351 | [3] | C1d1 |
| HM107352 | [3] | C1d1 |
| HM107353 | [3] | C1d1 |
| HM107354 | [3] | C1d1 |
| **Accession** | **Reference** | **Subhaplogroup** |
| HM107355 | [3] | C1d1 |
| HM107356 | [3] | C1d1 |
| HM107357 | [3] | C1d1 |
| HM107358 | [3] | C1d1 |
| HM107359 | [3] | C1d1 |
| HM107360 | [3] | C1d1 |
| HM107361 | [3] | C1d1 |
| HM107362 | [3] | C1d1 |
| HM107363 | [3] | C1d1 |
| HM107364 | [3] | C1d1 |
| HM107365 | [3] | C1d1 |
| HM107367 | [3] | C1d1 |
| HM107368 | [3] | C1d1 |
| HQ012235 | [4] | C1d1 |
| HQ012237 | [4] | C1d1 |
| HQ012238 | [4] | C1d1 |
| HQ012242 | [4] | C1d1 |
| HQ012244 | [4] | C1d1 |
| JQ701741 | [5] | C1d3 |
| KP017255 | This work | C1d3 |
| KP017256 | This work | C1d3 |
| KP017257 | This work | C1d3 |
| KP017258 | This work | C1d3 |
| HM107306 | [3] | C1d* |
| HM107307 | [3] | C1d* |
| HM107308 | [3] | C1d* |
| HM107309 | [3] | C1d* |
| HM107310 | [3] | C1d* |
| HM107311 | [3] | C1d* |
| HM107312 | [3] | C1d* |
| HM107313 | [3] | C1d* |
| HM107314 | [3] | C1d* |
| HM107315 | [3] | C1d* |
| HM107316 | [3] | C1d* |
| HM107317 | [3] | C1d* |
| HQ012234 | [4] | C1d* |
| HQ012239 | [4] | C1d* |
| HQ012240 | [4] | C1d* |
| HQ012241 | [4] | C1d* |
| HQ012243 | [4] | C1d* |
| JX413049 | [6] | C1d* |
| JX413050 | [6] | C1d* |
| JX669313 | Tito et al., unpublished | C1d* |
| JX669333 | Tito et al., unpublished | C1d* |

1. **Rho-based TMRCA estimates**

The ρ-statistic [7,8] to the putative ancestral haplotype of C1d (C1+194T+16051G) was calculated for the entire set of sequences listed in section 1. For the Uruguayan set of C1d3 sequences, the corresponding ρ-statistic was calculated to the putative ancestral haplotype of C1d3 (C1d+12378T+16140C+16288C). In both cases, insertions, deletions, and mutations 16182C, 16183C, 16194C and all variants in position 16519 were ignored. For the 95% confidence interval, ρ was recalculated on 10,000 boostrap samples of the sequences and percentiles 2.5 and 97.5 were obtained. The age estimate was then carried out using the whole-genome mitochondrial substitution rate corrected for time depth published by [9], which accounts for the slight positive skew in the interval (Table 1). All calculations were carried out using the *ape* package [10] in R 3.1.2 [11].

| **Haplogroup** | **95% CI Lower bound** | **Estimate** | **95% CI Upper bound** |
| --- | --- | --- | --- |
| **C1d** | 19180.8 | 20633.8 | 22130.6 |
| **C1d3** | 5747.9 | 8973.9 | 12261.4 |

Table A. 95% confidence interval for the TMRCA estimates (years) of C1d and C1d3 using ρ.

1. **Bayesian TMRCA estimates**

Using the sequences listed in section 1, a TMRCA estimate of C1d and C1d3 was calculated through a Bayesian skyline plot (BSP [12]) using BEAST2 [13]. The estimate was carried out using the whole-genome mitochondrial substitution rate of 1.665x10^-8^ per nucleotide per year, assuming a strict molecular clock and the TN93 substitution model [14]. Three clades were set as priors: C1d1 (C1d+7697A), C1d3 (C1d+12378T+16140C+16288C) and C1d* (*sensu* [3], which includes C1d3). C1d1 and C1d3 were set as monophyletic, while C1d* was not. The chain length was set to 2x10^7^ with a burn-in length of 1x10^6^.

| **Haplogroup** | **95% CI Lower bound** | **Estimate** | **95% CI Upper bound** |
| --- | --- | --- | --- |
| **C1d** | 18347.9 | 22032.7 | 26335.5 |
| **C1d3** | 4837.4 | 9487.3 | 14303.7 |

Table B. 95% confidence interval for the TMRCA estimates (years) of C1d and C1d3 using BSP.

1. **Calibrated TMRCA estimate**

For a control of the ratio of the TMRCA estimates of C1d3 and C1d, which in turn would allow us to estimate not the exact time but rather the proportion of time elapsed between the origin of pan-American haplogroup C1d and the origin of the strongly local subhaplogroup C1d3, a third time estimate was made in BEAST2, this time for C1d3 calibrated using a previous TMRCA estimate of 16,200 ± 1,100 years [3]. In terms of the ratio between the age of C1d3 and C1d, the point estimate of the age of C1d3 is highly consistent using all three methods, setting the age of C1d3 at 43% to 44% of the age of C1d.

| **Haplogroup** | **95% CI Lower bound** | **Estimate** | **95% CI Upper bound** |
| --- | --- | --- | --- |
| **C1d (pre-set)** | 13900 | 16200 | 18200 |
| **C1d3** | 3416.8 | 7274.8 | 10200 |

Table C. 95% confidence interval for the TMRCA estimate (years) of C1d3 calibrated with a previous C1d age estimate.

It is worth noting that the radiocarbon date of CH2D01-20 was not included as a “tip date” in the Bayesian estimate analysis as it yielded nonsensical results.

**References**

1. Just RS, Diegoli TM, Saunier JL, Irwin JA, Parsons TJ. Complete mitochondrial genome sequences for 265 African American and U.S. “Hispanic” individuals. Forensic Science International: Genetics. 2008; 2: e45-e48.

2. Fagundes NJR, Kanitz R, Eckert R, Valls ACS, Bogo MR, Salzano FM, et al. Mitochondrial Population Genomics Supports a Single Pre-Clovis Origin with a Coastal Route for the Peopling of the Americas. Amer J Hum Genet. 2008; 82: 583-592.

3. Perego UA, Angerhofer N, Pala M, Olivieri A, Lancioni H, Kashani BH, et al. The initial peopling of the Americas: A growing number of founding mitochondrial genomes from Beringia. Genome Res. 2010; 20: 1174-1179.

4. Kumar S, Bellis C, Zlojutro M, Melton PE, Blangero J, Curran JE. Large scale mitochondrial sequencing in Mexican Americans suggests a reappraisal of Native American origins. BMC Evol Biol. 2011; 11: 293.

5. Sans M, Figueiro G, Hidalgo PC. A New Mitochondrial C1 Lineage from the Prehistory of Uruguay: Population Genocide, Ethnocide and Continuity. Hum Biol. 2012; 84: 287-305.

6. de Saint Pierre M, Gandini F, Perego UA, Bodner M, Gómez-Carballa A, Corach D, et al. Arrival of Paleo-Indians to the Southern Cone of South America: New Clues from Mitogenomes. PLoS ONE. 2012; 7: e51311.

7. Forster P, Harding R, Torroni A, Bandelt HJ. Origin and evolution of Native American mtDNA variation: a reappraisal. Amer J Hum Genet. 1996; 59: 935-945.

8. Morral N, Bertranpetit J, Estivill X, Nunes V, Casals T, Gimenez J, et al. The origin of the major cystic fibrosis mutation (ΔF508) in European populations. Nat Genet. 1994; 7: 169-175.

9. Soares P, Ermini L, Thomson N, Mormina M, Rito T, Röhl A, et al. Correcting for Purifying Selection: An Improved Human Mitochondrial Molecular Clock. Amer J Hum Genet. 2009; 84: 740-759.

10. Paradis E, Claude J, Strimmer K. APE: analyses of phylogenetics and evolution in R language. Bioinformatics. 2004; 20: 289-290

11. R Core Team R: A language and environment for statistical computing. Vienna: R Foundation for Statistical Computing; 2014.

12. Drummond AJ, Rambaut A, Shapiro B, Pybus OG. Bayesian Coalescent Inference of Past Population Dynamics from Molecular Sequences. Mol Biol Evol. 2005; 22: 1185-1192.

13. Bouckaert R, Heled J, Kühnert D, Vaughan T, Wu C-H, Xie D, et al. BEAST 2: A Software Platform for Bayesian Evolutionary Analysis. PLoS Comput Biol. 2014; 10: e1003537.

14. Tamura K, Nei M. Estimation of the number of Nucleotide Substitutions in the Control Region of Mitochondrial DNA in Humans and Chimpanzees. Mol Biol Evol. 1993; 10: 512-526.
