# Supplementary material for: A South American Prehistoric Mitogenome: Context, Continuity, and the Origin of Haplogroup C1d
Source: PLoS One. 2015 Oct 28;10(10):e0141808. doi: 10.1371/journal.pone.0141808 (PMC4625051; doi:10.1371/journal.pone.0141808)
Supplement: S3 Text — (DOC) [file pone.0141808.s005.doc]

**S3 Text. Complete list of mutations found in the Uruguayan samples, respective to the revised Cambridge Reference Sequence (rCRS)**

**Complete C1d3 Uruguayan mitogenomes:**

**CH2D01-20 (ancient) (KP017255) and B11 (JQ701741):** 73G-194T-249d-263G-290d-291d-315.1C-489C-522d-523d-750G-1438G-2706G-3552A-4715G-4769G-7028T-7196A-8584A-8701G-8860G-9540C-9545G-10398G-10400T-10873C-11719A-11914A-12378C-12705T-13263G-14318C-14766T-14783C-14992C-15043A-15301A-15326G-15487T-15662G-16051G-16140C-16223T-16288C-16298C-16325C-16327T-16422C-16519C

**KC018 (KP017256) and M22 (KP017258):** 73G-194T-249d-263G-290d-291d-315.1C-489C-507C-522d-523d-750G-1438G-2706G-3552A-4715G-4769G-7028T-7196A-8584A-8701G-8860G-9540C-9545G-10398G-10400T-10873C-11719A-11914A-12378C-12705T-13263G-14318C-14766T-14783C-15043A-15301A-15313C-15326G-15487T-16051G-16209C-16140C-16223T-16288C-16298C-16325C-16327T-16400T-16422C-16519T@

**KC208 (KP017257):** 73G-194T-249d-263G-290d-291d-315.1C-489C-522d-523d-750G-1438G-2706G-3552A-4715G-4769G-7028T-7196A-8474T-8584A-8701G-8860G-9540C-9545G-10365A-10398G-10400T-10873C-11719A-11914A-12378C-12705T-13263G-14318C-14766T-14783C-15043A-15301A-15326G-15487T-16051G-16140C-16223T-16288C-16298C-16325C-16327T-16519C

**Table:**

| **Position** | **rCRS** | **CH2D01-20** | **B11** | **KC018** | **M22** | **KC208** |
| --- | --- | --- | --- | --- | --- | --- |
| **73** | **A** | G | G | G | G | G |
| **194** | **C** | T | T | T | T | T |
| **249** | **A** | del | del | del | del | del |
| **263** | **A** | G | G | G | G | G |
| **290-291** | **AA** | del | del | del | del | del |
| **315** |  | ins C | ins C | ins C | ins C | ins C |
| **489** | **T** | C | C | C | C | C |
| **507** | **T** | T | T | C | C | T |
| **522-523** | **CA** | del | del | del | del | del |
| **750** | **A** | G | G | G | G | G |
| **1438** | **A** | G | G | G | G | G |
| **2706** | **A** | G | G | G | G | G |
| **3552** | **T** | A | A | A | A | A |
| **4715** | **A** | G | G | G | G | G |
| **4769** | **A** | G | G | G | G | G |
| **7028** | **C** | T | T | T | T | T |
| **7196** | **C** | A | A | A | A | A |
| **8474** | **C** | C | C | C | C | T |
| **8584** | **G** | A | A | A | A | A |
| **8701** | **A** | G | G | G | G | G |
| **8860** | **A** | G | G | G | G | G |
| **9540** | **T** | C | C | C | C | C |
| **9545** | **A** | G | G | G | G | G |
| **10365** | **G** | G | G | G | G | A |
| **10398** | **A** | G | G | G | G | G |
| **10400** | **C** | T | T | T | T | T |
| **10873** | **T** | C | C | C | C | C |
| **11719** | **G** | A | A | A | A | A |
| **11914** | **G** | A | A | A | A | A |
| **12378** | **C** | T | T | T | T | T |
| **Position** | **rCRS** | **CH2D01-20** | **B11** | **KC018** | **M22** | **KC208** |
| **12705** | **C** | T | T | T | T | T |
| **13263** | **A** | G | G | G | G | G |
| **14318** | **T** | C | C | C | C | C |
| **14766** | **C** | T | T | T | T | T |
| **14783** | **T** | C | C | C | C | C |
| **14992** | **T** | C | C | T | T | T |
| **15043** | **G** | A | A | A | A | A |
| **15301** | **G** | A | A | A | A | A |
| **15313** | **T** | T | T | C | C | T |
| **15326** | **A** | G | G | G | G | G |
| **15487** | **A** | T | T | T | T | T |
| **15662** | **A** | G | G | A | A | A |
| **16051** | **A** | G | G | G | G | G |
| **16140** | **T** | C | C | C | C | C |
| **16209** | **T** | T | T | C | C | T |
| **16223** | **C** | T | T | T | T | T |
| **16288** | **T** | C | C | C | C | C |
| **16298** | **T** | C | C | C | C | C |
| **16325** | **T** | C | C | C | C | C |
| **16327** | **C** | T | T | T | T | T |
| **16400** | **C** | C | C | T | T | C |
| **16422** | **T** | C | C | C | C | T |
| **16519** | **T** | C | C | T | T | C |
